# Species- and Age-Dependent Prenyllipid Accumulation in *Hypericum* Species’ Leaves

**DOI:** 10.3390/plants14142239

**Published:** 2025-07-20

**Authors:** Danija Lazdiņa, Ieva Miķelsone, Inga Mišina, Krists Dukurs, Ana M. Benítez-González, Carla M. Stinco, Antonio J. Meléndez-Martínez, Paweł Górnaś

**Affiliations:** 1 Institute of Horticulture, Graudu 1, LV-3701 Dobele, Latvia; danija.lazdina@lathort.lv (D.L.); ieva.mikelsone@lathort.lv (I.M.); inga.misina@lathort.lv (I.M.); krists.dukurs@lathort.lv (K.D.); 2 Laboratory of Food Color and Quality, Department of Nutrition and Food Science, Faculty of Pharmacy, University of Seville, 41012 Seville, Spain; abenitez@us.es (A.M.B.-G.); cstinco@us.es (C.M.S.); ajmelendez@us.es (A.J.M.-M.)

**Keywords:** herb, Hypericaceae, aerial part, phytomedicine, bioactive phytochemical, vitamin E

## Abstract

Carotenoid, chlorophyll and tocochromanol biosynthesis and accumulation are interrelated and age-dependent in plants. Model plants produce tocopherols, but do not produce significant amounts of tocotrienols; consequently, the regulation of tocotrienol biosynthesis in plants has been scarcely studied. The *Hypericum* genus produces a variety of prenyllipids naturally in all parts of the plant, allowing for a glimpse into the relationship between them without genetic or other interference. Consequently, five *Hypericum* species’ leaves of different ages were investigated—*H. androsaemum*, *H. pseudohenryi*, *H. hookerianum*, *H. patulum* and one hybrid *H.* × *inodorum* (*H. androsaemum* × *H. hircinum*). The leaves contained predominantly α-tocopherol, γ-tocotrienol and δ-tocotrienol (30.9–212.8, 8.13–22.43 and 1.87–20.8 mg 100 g^−1^, respectively). Higher quantities of tocochromanols, a lower chlorophyll content and a higher *a*/*b* ratio were observed in the bottom (older) leaves. The predominant carotenoids were lutein (semi-quantitative) and β-carotene (7.60–28.63 and 2.33–12.43 mg 100 g^−1^, respectively). Carotenoid contents were lower in bottom leaves than in middle or top leaves, and the highest carotenoid content was observed in *H. hookerianum* and *H. patulum*. Leaf tocopherol, tocotrienol, chlorophyll and carotenoid accumulation were section and leaf age-dependent, and distinct relationships can be observed between the accumulation of some prenyl lipids, but not others.

## 1. Introduction

Now recognized as a separate plant family, Hypericaceae used to be classified as a subfamily of the Clusiaceae family until 2003, when the Angiosperm Phylogeny Group APG II system classified it as a separate family [1]. The *Hypericum* genus is one of six genera in the Hypericaceae family. It is collectively referred to as St. John’s wort or goatweed, and some members are referred to as tutsan. The genus includes mostly perennial flowering shrubs and herbs. The aerial, flowering parts of several species in the *Hypericum* genus are collected and used for phytomedicine. Although exact data are not available, the number and array of *Hypericum* products on the market demonstrates the economic viability and established agricultural practices of the genus. Extraction techniques include water extracts (tea), hydroalcoholic extracts [2,3] and oil macerations [4]. The primary compounds of concern are naphthodianthrones (hypericin, pseudohypericin) and prenylated phloroglucinols (hyperforin, adhyperforin), specific to the genus, and other phenolic compounds such as flavonoids [2]. Naphthodianthrones and acylphloroglucinols are considered characteristic compounds in the *Hypericum* genus, although the presence and content can vary significantly between species, with *H. perforatum* being the richest in both, while *H. hircinum* contains neither or trace amounts [5]. Other species contain varying amounts of flavonoids, catechins and phenolic acids, but their contents do not necessarily directly correlate with the antioxidant activity of plant extracts, indicating the presence of alternate antioxidant molecules [5].

Tocochromanols and carotenoids are lipophilic antioxidants present in all plant parts. Tocopherols and tocotrienols protect the plant cellular membrane and reserve lipids against peroxidation and act as signaling compounds [6]. The biosynthesis of both uses the shikimate and MEP pathway. Tocochromanols are made up of a chromane ring with specific methyl group placement and a lipophilic isoprenoid tail. The lipophilic tail determines the tocochromanol type—tocopherols have a saturated tail, while tocotrienols, tocodienols and tocomonoenols have three, two and one saturated bond in the tail, respectively. The placement of methyl groups on the chromane ring determines the tocochromanol homologue. Tocopherols and tocotrienols have common biosynthesis pathways, but use different precursor compounds—tocotrienol biosynthesis require homogentisate to produce geranylgeranyl pyrophosphate [7], the direct tocotrienol precursor, while phytyl pyrophosphate (PPP), used for the tocopherol tail, can be produced from either homogentisate or phytol from degraded chlorophyll [8]. Tocopherols are more common and their presence in plants is far more studied than that of tocotrienols, the next most recognized group of tocochromanols. There is a preference for accumulating α-T in the leaves, while other parts of plants tend to be richer in γ-T [9]. The literature generally indicates that higher levels of tocotrienols are found in the seeds and oils of certain plant species [10,11]. Initially, it was believed that tocotrienols were mainly associated with monocotyledonous plants, particularly those belonging to the Poaceae [6,9] and Arecaceae [12] families. However, more recent research has revealed that dicotyledonous plants, including seeds from families such as Apiaceae [13,14], Ericaceae [10,15] and Vitaceae [10] also contain significant quantities of tocotrienols. Additionally, emerging evidence highlights that the leaves of certain genera, such as *Clusia* and *Hypericum*, may serve as promising and underexplored sources of these bioactive compounds [16,17,18]; therefore, it is worth taking a closer look at these genera, especially *Hypericum*. While several reports have recently been published on the presence of tocopherols and tocotrienols in *Hypericum* leaves and several plant organs of *Hypericum perforatum* [17,19,20], these did not investigate the relationship between tocochromanols and other common minor lipids—chlorophyll, which shares chemical precursors with tocochromanols, and carotenoids, which share precursors and physiological functions.

While tocochromanols contain an isoprenoid chain, carotenoids are wholly isoprenoid compounds and contain several unsaturated bonds; all are tetraterpene derivatives. The isoprenoid chain is produced by condensing two GGPP molecules into phytoene, from which carotenes and xanthophylls are produced through successive desaturation, cyclization and epoxidation [21]. More total carotenoids are accumulated in leaves under high-light conditions than in the shade, and their composition is lighting-dependent—some xanthophylls may only be produced under sufficient light [22]. Carotenoids are health-promoting compounds that contribute to reduce the risk of cancer, cardiovascular disease and eye, bone and skin conditions. They are also associated with neurological and metabolic benefits [23]. Apart from providing health benefits and (some of them) being precursors of vitamin A, carotenoids are colorants and can protect foods from oxidation and provide cosmetic benefits, hence their versatility in a variety of products for the agri-food, pharma and cosmetic industries [24].

**Figure 1:**
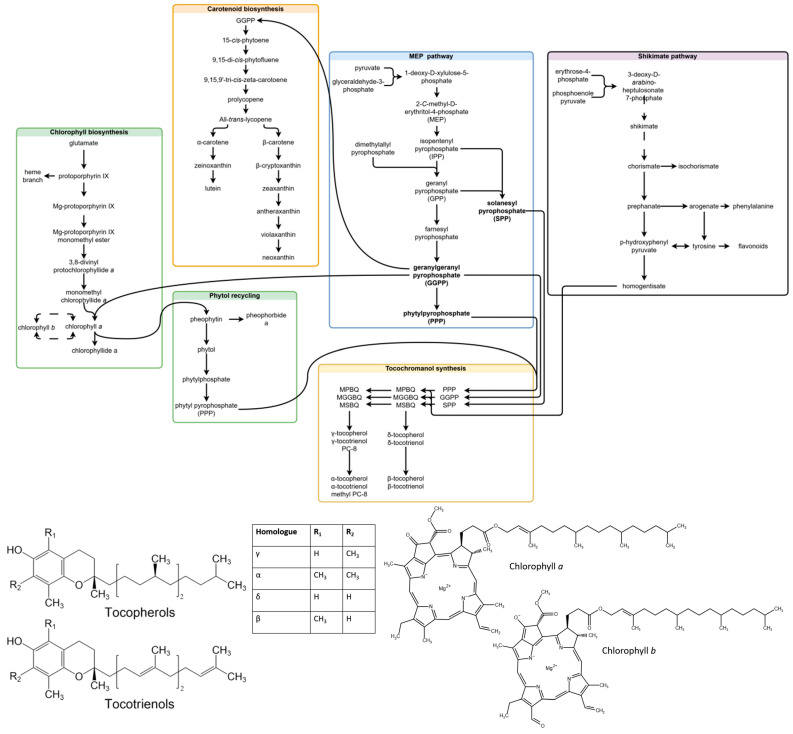

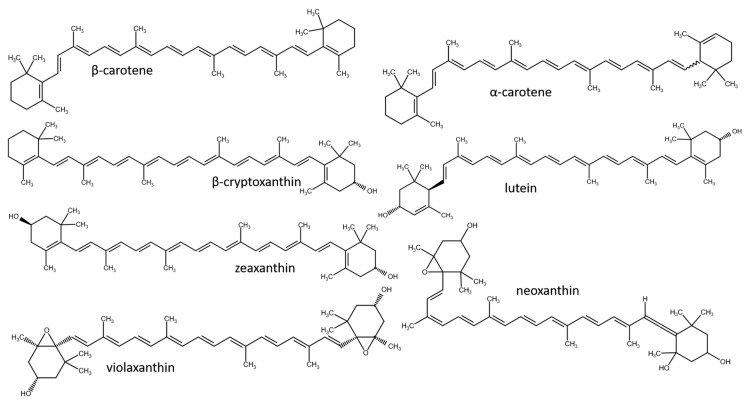
Related steps in chlorophyll, carotenoid and tocochromanol biosynthesis, and molecular structures of the analyzed compounds. Some unrelated steps are shown, and the simplified transformation of contents (missing steps) is represented by dashed lines. The scheme is a combination of biosynthesis pathways published in existing articles [7,25,26,27,28,29]. Abbreviations not deciphered in chart: MPBQ, 2-methyl-6-phytyl-1,4-benzoquinol; MGGBQ, 2-methyl-6-geranylgeranyl-1,4-benzoquino; MSBQ, 2-methyl-6-solanesyl-1,4-benzoquinol; DMPBQ, 2,3-dimethyl-6-phytyl-1,4-benzoquinol; DMGGBQ, 2,3-dimethyl-6-geranylgeranyl-1,4-benzoquinol; DMSBQ, 2,3-dimethyl-6-solanesyl-1,4-benzoquinol.

Chlorophylls are a type of green tetrapyrrole pigment involved in photosynthesis. Two types of chlorophyll are produced in plants—chlorophyll *a* (Chl *a*) and chlorophyll *b* (Chl *b*). Both consist of a tetrapyrrole macrocycle with Mg^+2^ at its core, a phytol chain and an additional fifth ring structure. Chlorophylls are differentiated by the chemical substituent at C7—a methyl group in Chl *a*, and a formyl group in Chl *b*. Chl *a* and Chl *b* can be converted into each other directly or indirectly (through chlorophyllides) in the chlorophyll cycle, and the indirect route is considered more common [25]. Chlorophylls (*a* + *b*) are accumulated under high-light conditions, and the *a*/*b* ratio is increased [22]. The basic common precursors and steps in chlorophyll, tocochromanol and carotenoid biosynthesis, as well as the compounds analyzed in the study are provided in Figure 1.

Besides *H. perforatum*, a number of *Hypericum* species are cultivated as ornamental plants, including hardy shrubs, which produce a significant amount of biomass each year. The branches and leaves of the shrubs are typically cut each year for wintering. Considering recent reports on diverse tocochromanol profiles in *Hypericum* leaves [16,17,18], the investigation of the cut material presents opportunities for the study of leaf tocochromanol metabolism in a plant that produces tocotrienols naturally, as well as a potential raw material for tocopherol and tocotrienol extraction. For these reasons, the present study firstly investigates the tocochromanol, chlorophyll and carotenoid contents of *Hypericum* leaves from branches cut for wintering and secondly aims to provide initial reports on the relationship between prenyllipid accumulation in *Hypericum* leaves, since existing literature on other plant species has demonstrated that both leaf age and position influence the biosynthesis and accumulation of tocochromanols [30,31].

## 2. Materials and Methods

### 2.1. Reagents

Ethanol, methanol, ethyl acetate, *n*-hexane (HPLC grade), pyrogallol, sodium chloride and potassium hydroxide (reagent grade) were received from Sigma-Aldrich (Steinheim, Germany). Ethanol (96.2%), for the lab-scale extraction of bioactive compounds from *H. perforatum*, was purchased from SIA Kalsnavas Elevators (Jaunkalsnava, Latvia) and was used for the preparation of different hydroethanolic solutions. Standards of tocopherol homologues (α, β, γ and δ) (≥98%, HPLC) were purchased from Extrasynthese (Genay, France), and tocotrienol homologues (α, β, γ and δ) (≥98%, HPLC) and β-carotene (≥95%, HPLC) standards were purchased from Cayman Chemical (Ann Arbor, MI, USA), respectively.

### 2.2. Plant Material

The leaves of five *Hypericum* genus species were harvested in the garden of the Institute of Horticulture, Dobele, Latvia (GPS location: N: 56°36′39″ E: 23°17′50″) from plants planted three years prior. The plants were grown from seedlings in an open field using seeds received from botanical gardens—*Hypericum androsaemum* and *Hypericum × inodorum* (Plant World, St. Marychurch Rd, UK), *Hypericum pseudohenryi* (Botanical Garden of Marie Curie-Sklodowska University in Lublin, Poland), *Hypericum hookerianum* (Botanical Garden of the Faculty of Science, Masaryk University, Brno, Czech Republic) and *Hypericum patulum* (Tallinn Botanic Garden, Estonia). In the middle of October 2024, three plants from each species (biological replicates, *n* = 3) were randomly selected and cut off close to the soil line. Leaves from each plant were analyzed individually and treated as biological replicates in the experiment. Each plant had a minimum of 20 branches, from which 5 to 6 branches were randomly selected for sampling. For each plant, leaves were collected by separating 2–3 leaf pairs from each stem section—bottom, middle and top, as shown in the Supplementary Materials (Figures S1–S5). The plant branches were collected during the morning hours (9:00–10:00 local time) and transported to the lab in dark polyethylene bags within 15 min. All leaves were separated within 30–60 min after delivery to the lab. Separate leaves were immediately frozen in the freezer at −80 ± 2 °C for 2 h and then transferred for freeze-drying (Labconco, Kansas City, MO, USA) at a temperature of −51 ± 1 °C under a vacuum of 0.055–0.065 mbar for 72 h. On the same day (morning hours), after freeze-drying, leaves were milled using a MM400 ball mill (Retsch, Haan, Germany) to obtain a 5 µm final particle size. Moisture was determined gravimetrically. Sample preparation for tocochromanol, carotenoid and chlorophyll analysis were performed on the same day directly after milling the samples. The determination of phytochemicals was performed in the second part of the same day (chlorophyll by spectrometric method), and carotenoid and tocochromanol analyses were performed in the evening hours. All extractions were performed under limited light, low temperature and limited oxygen exposure.

### 2.3. Tocochromanol and Carotenoid Extraction

For tocopherols, tocotrienols and carotenoids, a single extraction procedure was proposed for the simultaneous isolation of phytosterols, tocopherols and lutein from soybeans [32]. The method reported by Slavin and Yu [32] was adapted and validated for the recovery of tocochromanols from apple seeds [33] and applied in the present study. The method consists of a semi-micro-saponification protocol followed by several extractions with a mixture of *n*-hexane:ethyl acetate (9:1, *v*/*v*), organic solvent evaporation, re-dissolving in 1 mL of ethanol and transfer to 2 mL glass vials for future LC analysis.

### 2.4. Tocopherol and Tocotrienol Analysis with RP-HPLC-FLD

A Shimadzu Nexera 40 Series HPLC system (Kyoto, Japan), with an RF-20Axs fluorescence detector (FLD) operated at the excitation wavelength of 295 nm and emission wavelength of 330 nm, was used for tocochromanol determination. Tocopherols and tocotrienols were separated using the Epic PFP-LB column (3 μm, 150 × 4.6 mm) (PerkinElmer, Waltham, MA, USA) secured with a PFP guard column (4 × 3 mm) (Phenomenex, Torrance, CA, USA). Analyses were performed under the following isocratic conditions: mobile phase, methanol:water in ratio of 91:9 (*v*/*v*); flow rate, 1.0 mL/min; column oven set at 40 °C; and total analysis run time of 13.5 min. Identification and quantification were performed based on tocopherol and tocotrienol standards and the obtained calibration curves. The tocopherol and tocotrienol stock solutions were appropriately diluted in ethanol to obtain an absorbance within the range of 0.2 to 0.5, ensuring linearity according to the Beer–Lambert law. Obtained standard solutions were determined spectrophotometrically using extinction coefficients for individual tocochromanols and the Beer–Lambert equation [34]. Details of the method of HPLC validation were provided earlier [3].

### 2.5. Carotenoid Content Semi-Quantitative Analysis

A Shimadzu Nexera 40 Series HPLC system (Kyoto, Japan) with an SPD-M40 diode-array detector (DAD) operated at a wavelength of 200–700 nm was used for carotenoid determination. Carotenoids were separated using the Kinetex PFP (pentafluorophenyl) column (5 μm, 250 × 4.6 mm) secured with a PFP guard column (4 × 3 mm) (Phenomenex, Torrance, CA, USA). The LC conditions for carotenoid determination were as follows: mobile phase, water (A) and methanol (B) with the following gradient: 0.01–3.5 min 87% B, 8.5 min 100% B, 10.5 min 100% B, 11.0 min 87% B; 15.0 min 87% B; flow rate, 1.0 mL/min; column oven temperature, 40 °C; and total analysis run time, 15 min. Due to budget limitations and the focus of the project on the presence of tocotrienols in the *Hypericum* genus, commercially available standards of carotenoids were not purchased. Instead, β-carotene was used for semi-quantification because it was in stock at the laboratory. Carotenoid identification was performed using isolated standards from various sources according to classical methodologies [35]: neoxanthin, violaxanthin and lutein from spinach; β-cryptoxanthin from mandarin flesh; zeaxanthin from goji berries; α-carotene from carrots; and a commercial β-carotene standard. UV spectra of the isolated standards and a representative chromatogram are provided in the Supplementary Materials. Due to the presence of some impurities in the isolated standards, quantifications were based on a calibration curve constructed for β-carotene at a detection wavelength of 450 nm. β-Carotene was dissolved in chloroform and subsequently diluted with hexane, 2-propanol and finally ethanol. The calibration curve was established within the range of 2–52 ng of β-carotene per injection. The application of a PFP column for carotenoid separation was based on a previous study, dictated, among other reasons, by the employment of less toxic solvents as a mobile phase [36]. The developed method was characterized by a short run time, but also lacked the separation of α-carotene and β-carotene. The separation of α-carotene and β-carotene was performed on the basis of their UV spectra. An example of an obtained chromatogram can be found in the Supplementary Materials (Figure S7).

### 2.6. Chlorophyll Extraction and Content Analysis

The chlorophyll was extracted from the leaf powder (0.100 ± 0.001g) using 96.2% ethanol (10 mL) and ultrasonic treatment. Briefly, the ultrasonic treatment was performed at 60 °C for 15 min in an ultrasonic bath Bandelin Sonorex RK 510 H (Bandelin electronic, Berlin, Germany) at a frequency of 35 kHz and nominal ultrasonic power of 160 W. The sample was mixed for 1 min on a vortex mixer REAX top (Heidolph, Schwabach, Germany) before and after the ultrasonic bath. Afterward, the sample was centrifuged at 11,000× *g* for 10 min at 21 °C to separate the supernatant from the plant material. The chlorophyll in the supernatant was measured at wavelengths of 665 nm and 649 nm using a UV-1800 spectrophotometer (Shimadzu, Kyoto, Japan), with the software UVProbe Spectrum (Shimadzu, Kyoto, Japan) for data processing. The content of chlorophyll *a* and *b* was calculated according to the following formula (µg mL^−1^) [37]:

Chlorophyll *a* = 13.70 × A_665_ − 5.76 × A_649_

Chlorophyll *b* = −7.60 × A665 + 25.8 × A649

### 2.7. Statistical Analysis

Two-way analysis of variance (ANOVA) was used to identify statistically significant differences between groups separated by qualitative variables (species and leaf age interaction). Tukey’s pairwise test was used to determine which qualitative variable groups were different from each other and to identify homogenous groups. Differences were considered statistically significant at *p* < 0.05. Spearman correlation coefficients (ρ) were used to determine the relationships between quantitative factors, as predicted by species, and chosen quantitative variables. Data exploration and statistical analysis were performed using R base and the open-source libraries stats, agricolae and GGally. The data were modified and visualized using the R packages tidyr, stringr, forcats, dplyr, ggplot2, ggthemes and patchwork, using RStudio version 2025.05.0+496 “Mariposa Orchid” Release (f0b76cc00df96fe7f0ee687d4bed0423bc3de1f8, 2025-05-04) for windows.

## 3. Results and Discussion

The leaves of five *Hypericum* species (including one hybrid) were collected and analyzed for tocochromanol, chlorophyll and carotenoid contents. Two of the species, *H. androsaemum* and *H. × inodorum*, belong to the *Androsaemum* section, while the rest belong to the *Ascyreia* section: *H. pseudohenryi*, *H. patulum* and *H. hookerianum*. The leaves of the *Androsaemum*-section species had started to senesce, while the rest were still green. This is unlikely to be a result of the plant type, since all of the species used are shrubby plants. Information about quantitative differences among the different secondary metabolites in the samples collected is summarized in the sections below. At this point, it is important to note that changes in phytochemical contents are subject to the age of the plant [38], age of the leaf [39,40] and climatic conditions [41,42,43,44].

### 3.1. Tocochromanols

**Figure 2:**
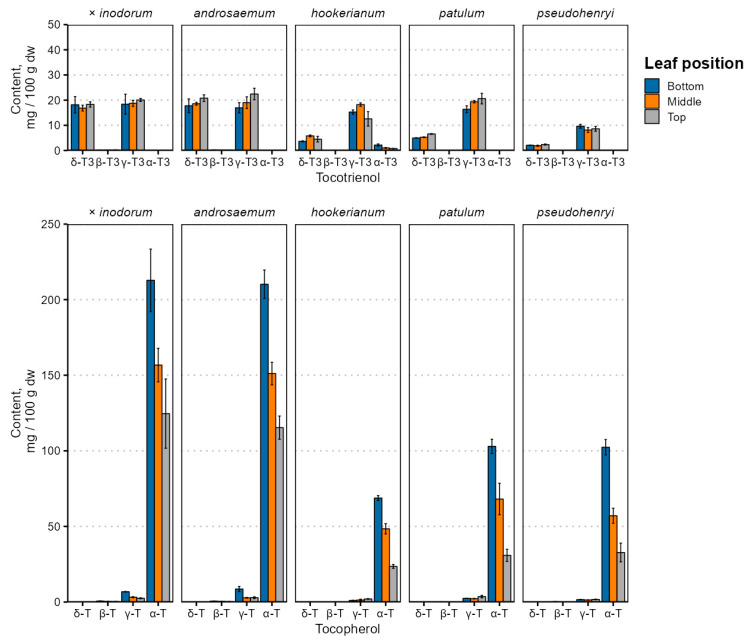
Tocochromanol content variation in *Hypericum* species’ leaves depending on leaf position. Data are presented as means ± standard deviations (*n* = 3).

Leaf age and species-dependent individual tocochromanol content changes are depicted in Figure 2, and the tocochromanol content is provided in Table S1.

Tocochromanol contents were highest in early (bottom) leaves in all species, and tocopherol content differed significantly between leaves of different positions (*p* < 0.001), while the tocotrienol content was not significantly affected by the leaf position (*p* = 0.72) and was relatively constant. Species and age interactions were strong for tocopherol (*p* = 0.0017) and tocotrienol (*p* = 0.0068) content. There were similarities with the tocopherol accumulation reported in *Phaseolus coccineus* (runner bean), which showed that the α-T content increased in leaves over a 50-day period [31]—leaves grown earlier in the vegetative period would have a higher tocopherol content.

By far, the major tocochromanol in *Hypericum* leaves was α-T, followed by γ- and δ-T3 in varying proportions, and some γ-T. While tocochromanol profiles are not widely studied in the *Hypericum* genus, a previous investigation of *H. perforatum* leaf methanol extracts observed α-T and smaller amounts of δ-T3 using ESI(+)-LC-MS [45]. The tocochromanol composition of the *Hypericum* leaves and the relatively high tocotrienol content are not entirely unprecedented. A relatively high tocotrienol proportion, specifically δ-T3, has been observed and reported in perilla (*Perilla frutescens*, Lamiaceae family) leaves, confirmed through LC-MS [46].

Tocochromanol contents, especially δ-T3 and α-T, were much higher in *H. × inodorum* and *H. androsaeumum*, which are closely related—*H. × inodorum* is a hybrid of *H. androsaemum* and *H. hircinum*. While *H. hircinum* leaves were not investigated in the present study, their leaf tocochromanols are composed almost entirely of α-T, and the total content was much lower—around 45 mg 100 g^−1^ dw [17]. The tocochromanol profiles of *H. × inodorum* and *H. androsaemum* are similar to those observed previously [18,47]. Individual tocochromanol contents within species did not appear to be strongly correlated with each other. Regarding major tocochromanols, there were statistically significant (*p* < 0.05), mild positive correlations between δ-T3 and γ-T3 contents in *H. androsaemum* (ρ = 0.71), *H. × inodorum* (ρ = 0.84) and *H. hookerianum* (ρ = 0.72). Statistically significant, strong correlations were observed between γ-T and α-T in *H. androsaemum* (ρ = 0.84), *H. × inodorum* (ρ = 0.92), *H. patulum* (ρ = 0.67) and *H. hookerianum* (ρ = −0.74). The tocotrienol and tocopherol content was negatively associated in *H. androsaemum* (ρ = 0.74) and *H. patulum* (ρ = −0.87). Negative correlations between tocotrienol and tocopherol contents may be explained by the upregulation of enzymes converting GGPP into MGGBQ for tocotrienol synthesis or converting GGPP into PPP for chlorophyll or tocopherol synthesis [48]. Distinct differences between the tocochromanol content and profile are rather common—the palm oil (*Elaeis guineensis*) tocochromanol content is significantly affected by both the population and geographic region [49], and the same is true for *H. perforatum* [50,51]. Like the geographic region, the phylogeny and species are major tocochromanol profile and content determinants in *Hypericum* [16,17] and other genera, like the *Prunus* genus [52]. Even the plant variety has a significant effect on the tocochromanol content and biosynthesis [53]. However, metabolic regulation in the tocochromanol biosynthetic pathway has not been studied in the *Hypericum* genus and can be recommended in future studies.

Tocopherols (α-Ts) and plastoquinone-9 accumulate in fig and beech leaves during the vegetative period (increased 5.2 and 3.0 times, respectively) and their concentration is higher in older leaves (increased 18 and 20 times, compared to young leaves) [22]. In plants that accumulate tocotrienols in the leaves, such as *Vellozia gigantea* (Velloziaceae family), tocopherol and tocotrienol content varies significantly across the growing seasons [54]. In herbaceous plants such as *Phaseolus coccineus* (runner bean, Fabaceae family), the α-T, but not γ- or δ-T, content increases under unaltered conditions. In plant leaves grown in the shade, the α-T content decreased significantly by day 47 of 53 [31]. An increased tocochromanol content may be a protective mechanism in older leaves against increased oxidative stress regardless of senescence [55]. In *Manihot esculenta* (cassava, Euphorbiaceae family) leaves, the total tocopherol content increased over a 15-month period after planting [56].

### 3.2. Chlorophylls

**Figure 3:**
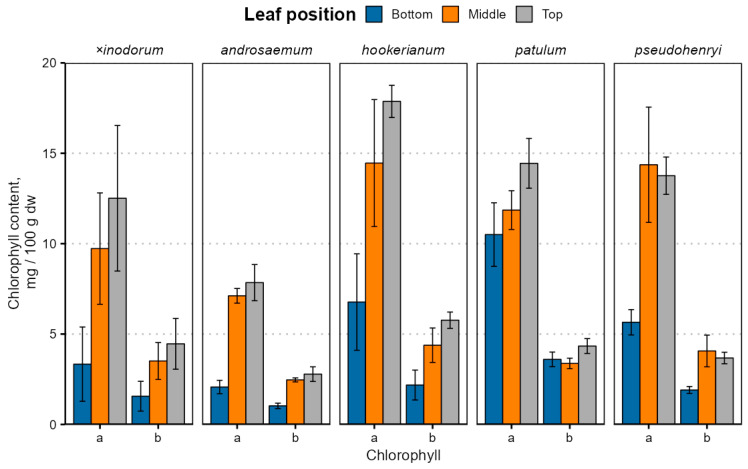
Chlorophyll content variation in *Hypericum* species’ leaves depending on the leaf position. Data are presented as means ± standard deviations (*n* = 3).

The leaf age- and species-dependent chlorophyll content variation is depicted in Figure 3; exact values are provided in Table S2.

Chlorophyll *a* and *b* contents were significantly different between leaves of different ages (*p* < 0.001) and species (*p* < 0.001) with a near-significant species–age interaction for chlorophyll *a* (*p* = 0.075) and a significant interaction for chlorophyll *b* (*p* = 0.016). The chlorophyll *a* and *b* content were similar in *H. patulum*, *H. pseudohenryi* and *H. hookerianum* (*p* > 0.05). The chlorophyll *b*, but not *a*, content was similar in *H. × inodorum*, *H*. *hookerianum*, *H. patulum* and *H. pseudohenryi* (*p* > 0.05). Only the chlorophyll *a* content was similar in *H. androsaemum* (*p* > 0.05), but, like chlorophyll *b*, it was also similar in *H. × inodorum* and *H. pseudohenryi* (*p* > 0.05). While early leaves were statistically significantly different from young and mature leaves (*p* < 0.001), the chlorophyll *a* content was similar in young and mature leaves (*p* = 0.076), but not chlorophyll *b* (*p* = 0.038). The chlorophyll content was relatively lower in *H. × inodorum* and *H. androsaemum*, largely due to distinct leaf senescence in the two species. The chlorophyll *a* and *b* content correlated positively in all species (ρ = 0.89 to 0.99), and *a*/*b* ratios were slightly lower (*p* < 0.001) in bottom leaves in most species, except *H. hookerianum*, but not significantly different between middle and top leaves (*p* = 0.69). The high standard deviation of chlorophyll observed in most of the leaf samples can be attributed to differences between sampled ages of branches. Among the various classes of prenyllipids, chlorophylls—and subsequently carotenoids—exhibit the lowest stability; as a result, their degradation is most pronounced during the later phases of plant maturation. Marked variations in chlorophyll content among the biological replicates were clearly observable via visual inspection, indicating noticeable differences in pigmentation between individual samples (Supplementary Materials, Figure S6).

The chlorophyll content was much lower than previously reported in *H. perforatum* leaves at 4 weeks old (212 mg 100 g^−1^ fw), grown hydroponically under white light [57], but the ratio of chlorophyll *a* and chlorophyll *b* was typical for most plants, including *Hypericum*. Decreased chlorophyll content in older (bottom) leaves is well-documented—tobacco leaf chlorophyll content decreases during natural leaf senescence [58], and basal (bottom) grapevine leaves have a lower chlorophyll content [59]. The chlorophyll content, especially chlorophyll *a*, decreases with stand age in the desert species *Haloxylon ammodendron* (Amaranthaceae or amaranth family), which negatively affects light use efficiency in older plants [60]. A slightly different trend has been observed in cassava leaves—chlorophyll *a* and *b* contents were slightly lower in the top leaves than middle and bottom [40]. It is important to note that different senescence-related genes are expressed in plant and leaves of different ages even at the same leaf senescence stage [61]. Besides natural senescence, cold may also cause chlorophyll contents to decrease [42]. Lower *a*/*b* ratios can be caused by low light reaching the bottom leaves or leaf aging, since lower *a*/*b* ratios have been observed in basal (bottom) grapevine leaves [59], as well as *H. perforatum* grown under red/blue light, compared to white light [57,62].

### 3.3. Carotenoids

**Figure 4:**
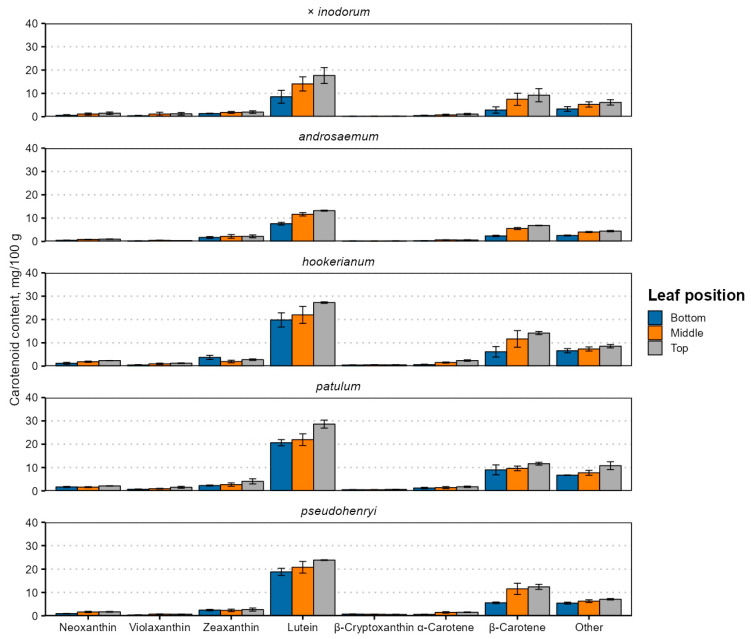
Carotenoid contents in senescent *Hypericum* species’ leaves of different position. Data are presented as means ± standard deviations of three replicates (*n* = 3).

Carotenoid contents were higher in younger leaves (Figure 4 and Table S3), and their content was generally strongly positively correlated, except for zeaxanthin and neoxanthin in *H. hookerianum* (ρ = −0.68) and α-carotene and β-cryptoxanthin in *H. pseudohenryi* (ρ = −0.68).

This is likely explained by the simultaneous upregulation of genes and enzymes involved in carotenoid biosynthesis [48]. The carotenoid content was higher in younger leaves. The main identified carotenoids in the leaves were lutein and β-carotene, in this order, regardless of their position. In total, up to seven carotenoids were identified. Apart from lutein and β-carotene, neoxanthin, violaxanthin, zeaxanthin (usually co-eluting with lutein), β-cryptoxanthin and α-carotene were present (Figure 4). The total carotenoid content ranged from 15.33 mg 100 g^−1^ in early (bottom) *H. androsaemum* leaves to 61.13 mg 100 g^−1^ in top (young) *H. patulum* leaves. The total carotenoid content was higher in *Ascyreia* species leaves than in *Androsaemum* section species leaves.

The results differ significantly from those observed in *H. perforatum* chloroform extracts, where the xanthophyll content was similar to the carotene content, and the α- and β-carotene content was much more similar, with the β-carotene content slightly higher than α-carotene [63]. However, the cited study investigated a different species, used the whole plant for extract preparation, as opposed to just the leaves, and used a different solvent than the present study, in addition to very roughly estimating individual carotenoid contents just from the absorbance at different wavelengths. The total carotenoid content in *H. perforatum* plants grown hydroponically under white light was 109 mg 100 g^−1^ in fresh leaves at 4 weeks old [57], much higher than in the present study. Unfortunately, detailed carotenoid profiles for *Hypericum* leaves or other organs are not available for comparison, and their investigations can be recommended in future studies on lipophilic *Hypericum* metabolites.

Lutein is typically accumulated in the highest concentration in plant leaves across a variety of species, families and plant types and is near-ubiquitous. In wide-scale screenings, its concentration can range from 16.4 mg 100 g^−1^ in *Tamarindus indica* (tamarind) leaves to 679 mg 100 g^−1^ dw in *Clitoria terneata* (butterfly pea) leaves [64]. Carotenoid contents in plant tissues are dependent on many variables, including the genotype, agronomic, climatic or physiological factors [65] and phylogeny. In the *Citrus* genus, the carotenoid profile is shaped mainly by the species and variety [66], and carotenoid profiles, related gene expression and deposit plastid structures differ significantly between different *Prunus* species and varieties [67]. A combination of these factors likely explains differences between *Hypericum* carotenoid profiles and contents and is a subject for future studies.

A small proportion of the other unidentified carotenoid sum may be antheraxanthin, which is an intermediary product between zeaxanthin and violaxanthin [21]. Alternatively, they may be products of carotenoid degradation, either as a result of normal plant metabolism or during sample preparation [68], or carotenoid derivatives, such as apocarotenoids [69], some of which act as chromophores.

The reduced carotenoid content in bottom leaves may be related to lower carotenoid biosynthesis and accumulation during the early months of growth or reduced light conditions resulting in low carotenoid and chlorophyll biosynthesis. In *Arabidopsis*, a model plant for studying lipid metabolism in plants, carotenoid accumulation decreases steadily with leaf age [40]. The same is true in tobacco leaves during natural senescence—older or earlier leaves at the bottom of the plant have lower carotenoid contents [39]. All investigated carotenoid contents were lower in the low-light stress group of *Festuca arundinacea* (tall fescue) leaves [41]. Existing research reports decreased chlorophyll *a*, chlorophyll *b* and carotenoid contents under red and blue lighting, an increased carotenoid content under blue lighting, compared to white light, and an increased net photosynthesis rate under blue light in hydroponically and foam-grown *H. perforatum* plants [57,62].

In *Manihot esculenta* (cassava, Euphorbiaceae family) leaves, carotenoid contents followed a slightly different trend—the carotenoid and chlorophyll content was lower in older plants (6 versus 12 months after planting), but was not distinctly more concentrated in higher leaves [38]. *H. androsaemum* and *H. × inodorum*, but not the other species, had senescent leaves. Like chlorophylls, the carotenoid content can decrease as a result of cold stress and acclimation [42], and their synthesis and accumulation cease upon leaf senescence [43]. Apart from leaf senescence, carotenoid biosynthesis is also affected by the temperature [44]. If the two *Androsaemum*-section species are less cold-hardy, carotenoid biosynthesis may have been downregulated regardless of leaf senescence.

### 3.4. Relationship Between Tocochromanol, Carotenoid and Chlorophyll Contents

While tocochromanols, carotenoids and chlorophylls serve different functions in plants, they have common biosynthetic pathways and precursor compounds. The biosynthesis upregulation of one group of compounds may necessitate the downregulation of others. To indirectly characterize the relationship among tocochromanol, carotenoid and chlorophyll biosynthesis, a correlation analysis was performed. Pair plots and Spearman corrrelation coefficients (ρ), along with the correlation significance, are provided in Figures S8–S10. A statistically significant correlation between tocotrienols and chlorophyll was only observed in *H. patulum* (ρ = 0.76) with chlorophyll a for δ-T3 (ρ = 0.78) and γ-T3 (ρ = 0.68). Tocopherol contents correlated negatively with the chlorophyll content in all species, especially chlorophyll *a* (ρ = −0.96 to −0.82), while chlorophyll *b* had a less pronounced correlation (ρ = −0.78 to −0.96). The negative correlation was most pronounced between α-T and chlorophyll *a* because α-T contents were the most significantly different between leaves of different ages. Other tocochromanol contents were relatively similar in leaves of different ages, resulting in a less significant correlation. Tocotrienols only had a significant correlation with carotenoids in *H. androsaemum* (ρ = 0.76) and *H. patulum* (ρ = 0.68). Tocopherols had a strong negative correlation with carotenoids in all species (ρ = −0.77 to −0.98). The ratios between analyzed metabolites are presented in Table 1.

Tocotrienol, tocopherol and the total tocochromanol ratio to total chlorophyll were lower in bottom leaves (*p* > 0.05), but similar in middle and top leaves (*p* < 0.001). The tocopherol and total tocochromanol ratio to total carotenoids was different between leaves of different ages (*p* < 0.05), but the tocotrienol ratio to total carotenoids was only different in bottom leaves (*p* < 0.01). Similarly, the carotenoid ratio to chlorophylls was similar in top and middle leaves (*p* = 0.85), but was generally higher in bottom leaves, except for *H. patulum*, in which the ratio did not differ significantly between leaves of different ages (*p* > 0.5), and the carotenoid content was diminished at the same rate as the chlorophyll content, whereas in other species, the carotenoid content was reduced more than chlorophyll.

It is important to note that while the plants were grown in the same soil and climatic conditions, they have different abilities to acclimate to those conditions, including frost, heat and sun exposure tolerance. While the effect of cold stress at 4 °C on *H. androsaemum* secondary metabolites (phenolic, anthraquinone and phloroglucinols compounds) was not pronounced, especially compared to tropical species like *H. canariense*, it did have an effect on the carotenoid content and antioxidant activity [70]. There is no available information on the effect of factors like cold stress on the tocochromanol profile in *Hypericum* plants at this time, and additional studies are necessary to discern between genetic and environmental effects on the tocochromanol profile. Of the investigated species, *H. androsaemum* is the only deciduous species, while the rest are semi-evergreen, depending on the climate, but this is only reflected in the lower chlorophyll content of the leaves.

## 4. Conclusions

Tocochromanol, carotenoid and chlorophyll accumulation in *Hypericum* leaves is species and leaf age-dependent. While tocopherol, carotenoid and chlorophyll contents are lower in older leaves, the tocotrienol content is lower in younger leaves. The present study provides insight into the content of and relationship between lipophilic metabolites in a species that has relatively high leaf tocotrienol contents and offers one of the first reports of the *Hypericum* leaf carotenoid profile. The results showed increased tocopherol contents in all species and increased tocotrienol contents in some species in the bottom leaves, while the chlorophyll and carotenoid contents were lower in older leaves. Considering the relatively high tocopherol and tocotrienol contents in the leaves, the *Hypericum* genus offers a unique opportunity for the study of the natural metabolic pathway of lipophilic antioxidants and pigments, which is only possible through genetic interference in current plant lipid metabolism model species.

Since the activity of the enzymes involved in the biosynthesis of the studied phytochemicals was not determined, it is not possible to make conclusions about their upregulation or downregulation depending on leaf age in *Hypericum* plants, and this study serves as an initial demonstration of the relationship between prenyl-lipid accumulation. Per general wintering advise, the plants were cut at the end of the vegetative season, but not during the vegetative season. As a result, the data reflect only phytochemical contents at one point in time in leaves of different heights and ages. As commented earlier, changes in phytochemical contents are subject to many variables besides the genotype, such as the age of the plant and the leaf, and their further study in the *Hypericum* genus is warranted. Changes in lipophilic secondary metabolite contents remain to be studied before, during and after blooming, as well as between growing seasons.

As a source of the analyzed prenyl-lipids, *Hypericum* leaves are not limited by biomass production and the concentration of these compounds in the biomass as it is by the presence of other compounds, which are co-extractable: phloroglucinols and naphtodianthrones, which will affect the biological properties of plant extracts and their applicability. However, some species produce little to no phloroglucinols and naphtodianthrones, like *H. hircinum*, which was investigated in the present study, while other *Hypericum* species produce no naphtodianthrones (*H. androsaemum* and *H. calycinum*) or no acylphloroglucinols (*H. montanum*) [5].

## Figures and Tables

**Table 1:** Lipophilic metabolite ratios in *Hypericum* leaves.

Species	Leaf Age	T3/Chl	T/Chl	TT/Chl	T3/Car	T/Car	TT/Car	Car/Chl
*Androsaemum* section								
*× inodorum*	Bottom	9.53 ± 5.48 ^a^	53.72 ± 22.69 ^a^	63.27 ± 27.68 ^a^	2.28 ± 0.98 ^a^	13.14 ± 3.50 ^a^	15.41 ± 4.32 ^a^	3.95 ± 0.85 ^abc^
	Middle	2.83 ± 0.74 ^b^	12.64 ± 2.59 ^b^	15.49 ± 3.30 ^b^	1.17 ± 0.27 ^bc^	5.2 ± 0.91 ^bc^	6.37 ± 1.17 ^bc^	2.42 ± 0.08 ^c^
	Top	2.43 ± 0.74 ^b^	8.23 ± 3.45 ^b^	10.65 ± 4.15 ^b^	1.02 ± 0.22 ^bcd^	3.44 ± 1.22 ^bcde^	4.46 ± 1.44 ^bcde^	2.35 ± 0.21 ^c^
*androsaemum*	Bottom	11.43 ± 1.96 ^a^	71.99 ± 10.87 ^a^	83.42 ± 11.95 ^a^	2.28 ± 0.18 ^a^	14.42 ± 1.82 ^a^	16.7 ± 1.79 ^a^	5.05 ± 1.03 ^a^
	Middle	3.93 ± 0.32 ^b^	16.16 ± 1.58 ^b^	20.11 ± 1.84 ^b^	1.48 ± 0.08 ^ab^	6.09 ± 0.70 ^b^	7.57 ± 0.77 ^b^	2.66 ± 0.17 ^c^
	Top	4.17 ± 0.91 ^b^	11.36 ± 2.37 ^b^	15.52 ± 3.25 ^b^	1.51 ± 0.09 ^ab^	4.12 ± 0.16 ^bcd^	5.63 ± 0.22 ^bcd^	2.75 ± 0.46 ^c^
*Ascyreia* section								
*hookerianum*	Bottom	2.60 ± 1.05 ^b^	8.63 ± 3.19 ^b^	11.26 ± 4.25 ^b^	0.55 ± 0.09 ^cd^	1.81 ± 0.26 ^de^	2.36 ± 0.35 ^de^	4.68 ± 1.22 ^ab^
	Middle	1.40 ± 0.36 ^b^	2.80 ± 0.98 ^b^	4.19 ± 1.35 ^b^	0.53 ± 0.07 ^cd^	1.06 ± 0.21 ^de^	1.59 ± 0.28 ^e^	2.61 ± 0.44 ^c^
	Top	0.77 ± 0.15 ^b^	1.09 ± 0.08 ^b^	1.84 ± 0.23 ^b^	0.3 ± 0.05 ^d^	0.43 ± 0.01 ^e^	0.73 ± 0.06 ^e^	2.52 ± 0.15 ^c^
*patulum*	Bottom	1.53 ± 0.15 ^b^	7.63 ± 1.39 ^b^	9.15 ± 1.54 ^b^	0.5 ± 0.03 ^cd^	2.49 ± 0.26 ^cde^	2.99 ± 0.28 ^cde^	3.05 ± 0.23 ^bc^
	Middle	1.63 ± 0.21 ^b^	4.68 ± 1.10 ^b^	6.31 ± 1.25 ^b^	0.54 ± 0.07 ^cd^	1.55 ± 0.43 ^de^	2.09 ± 0.5 ^d e^	3.04 ± 0.12 ^bc^
	Top	1.43 ± 0.21 ^b^	1.85 ± 0.22 ^b^	3.31 ± 0.41 ^b^	0.45 ± 0.07 ^d^	0.57 ± 0.09 ^e^	1.02 ± 0.15 ^e^	3.26 ± 0.17 ^bc^
*pseudohenryi*	Bottom	1.53 ± 0.12 ^b^	13.93 ± 1.87 ^b^	15.49 ± 1.94 ^b^	0.34 ± 0.04 ^cd^	2.99 ± 0.26 ^bcde^	3.33 ± 0.28 ^cde^	4.69 ± 0.87 ^ab^
	Middle	0.60 ± 0.17 ^b^	3.29 ± 0.79 ^b^	3.85 ± 0.95 ^b^	0.23 ± 0.05 ^d^	1.31 ± 0.26 ^de^	1.54 ± 0.31 ^e^	2.49 ± 0.23 ^c^
	Top	0.60 ± 0.00 ^b^	1.98 ± 0.22 ^b^	2.61 ± 0.24 ^b^	0.22 ± 0.02 ^d^	0.68 ± 0.10 ^e^	0.90 ± 0.12 ^e^	2.91 ± 0.18 ^c^

Data are presented as means ± standard deviations of three replications (*n* = 3). Letters denote statistically similar values within the column. Abbreviations: T3, tocotrienol; T, tocopherol; TT, total tocochromanols; Chl, total chlorophylls; Car, total carotenoids.

## Data Availability

The data used to support the findings of this study are available in the Supplementary Materials.

## Supplementary Materials

The following supporting information can be downloaded at: https://www.mdpi.com/article/10.3390/plants14142239/s1, Figure S1: *Hypericum androsaemum* and its top, middle, bottom leaves; Figure S2: *Hypericum × inodorum* and its top, middle, bottom leaves; Figure S3: *Hypericum pseudohendryi*; Figure S4: *Hypericum patulum*; Figure S5: *Hypericum hookerianum*; Figure S6: The powdered samples of top, middle, and bottom leaves of *Hypericum × inodorum*; Figure S7: Chromatogram of carotenoids separation of extracts from saponified samples of *H. patulum* top leaves by PFP column, together with the UV spectra of identified carotenoids and their retention times (top left corners); Figure S8: Spearman correlation between individual tocochromanols in *Hypericum* species’ leaves and tocochromanol content density plots; Figure S9: Spearman correlation between individual carotenoids in *Hypericum* species’ leaves and tocochromanol content density plots; Figure S10. Spearman correlation between total tocotrienols, total tocopherols, total tocochromanols, chlorophyll a and b, and total carotenoids in *Hypericum* species’ leaves and tocochromanol content density plots; Table S1: Tocochromanol content in *Hypericum* species’ leaves, mg 100 g^−1^ dw; Table S2: Chlorophyll content in *Hypericum* species’ leaves, mg 100 g^−1^ dw; Table S3: Carotenoid content in *Hypericum* species’ leaves, mg 100 g^−1^ dw.
