# Supplementary material for: Species- and Age-Dependent Prenyllipid Accumulation in Hypericum Species’ Leaves
Source: Plants (Basel). 2025 Jul 20;14(14):2239. doi: 10.3390/plants14142239 (PMC12298207; doi:10.3390/plants14142239)
Supplement: Supplementary file 1 [file plants-14-02239-s001.zip › plants-3732876-supplementary.pdf]

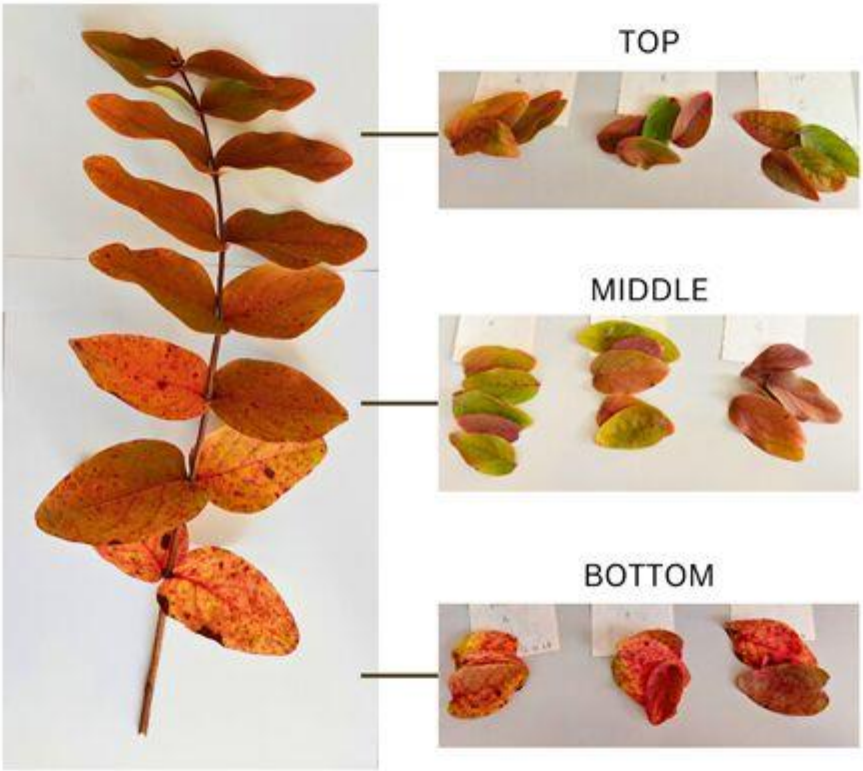

Figure S1. *Hypericum androsaemum* and its top, middle, bottom leaves

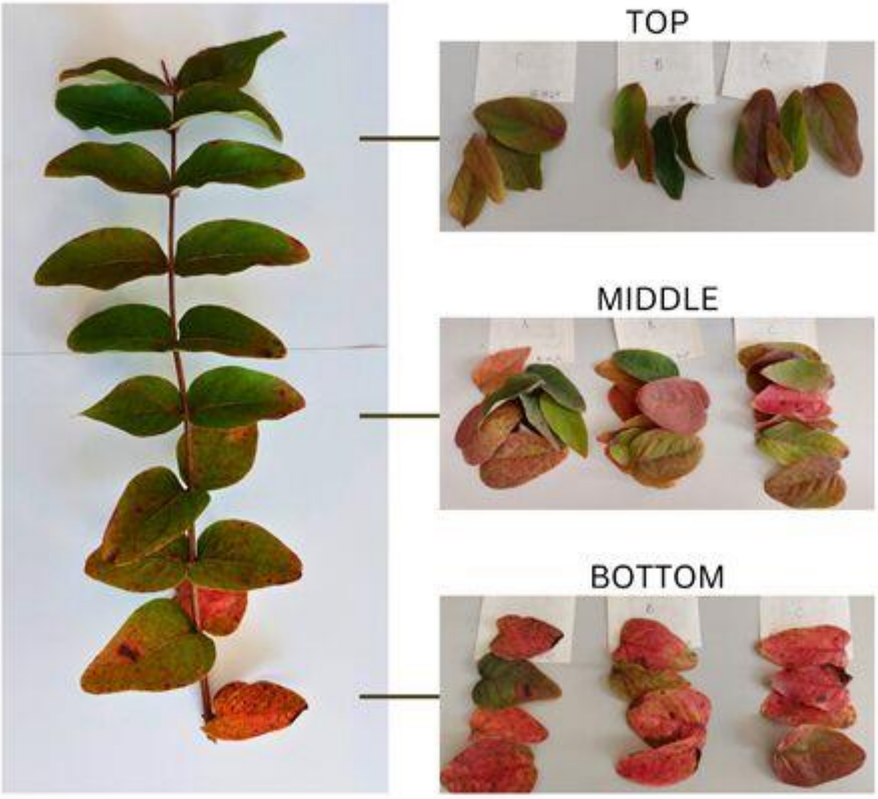

Figure S2. *Hypericum × inodorum* and its top, middle, bottom leaves

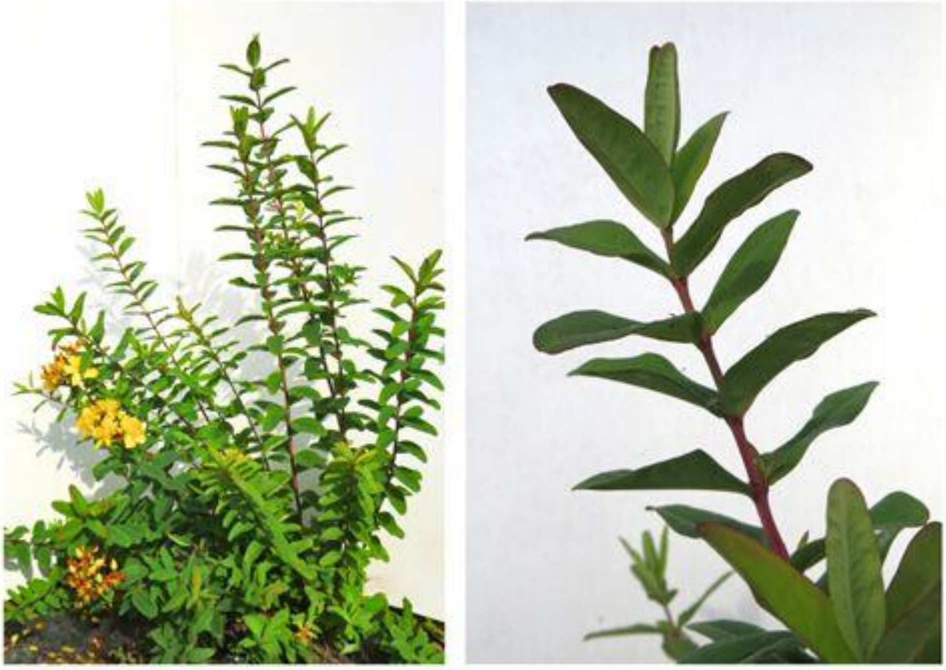

Figure S3. *Hypericum pseudohendryi*

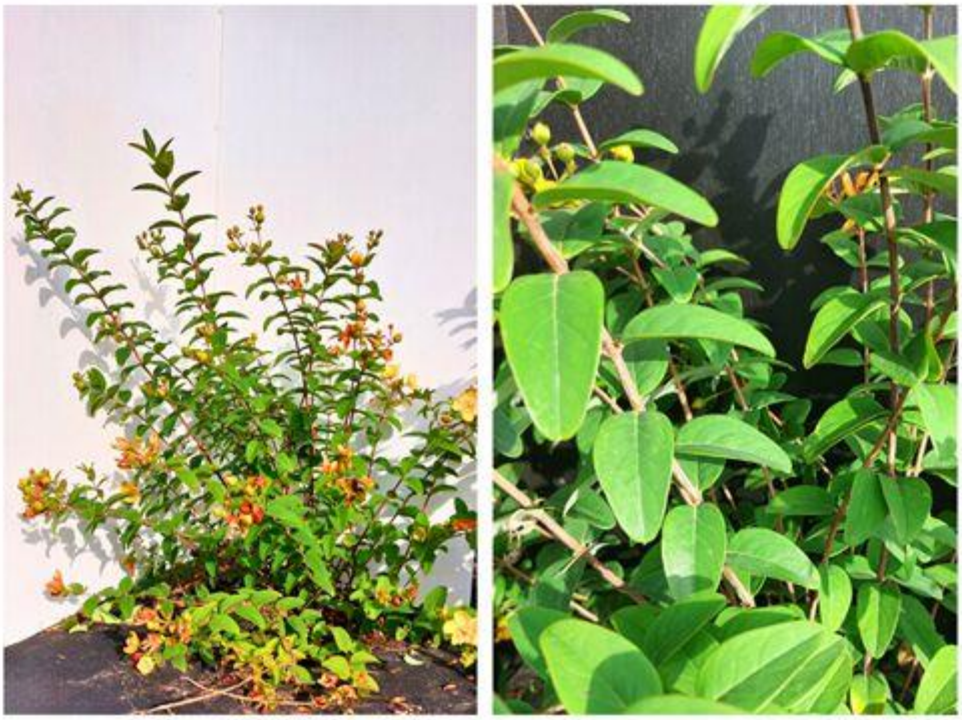

Figure S4. *Hypericum patulum*

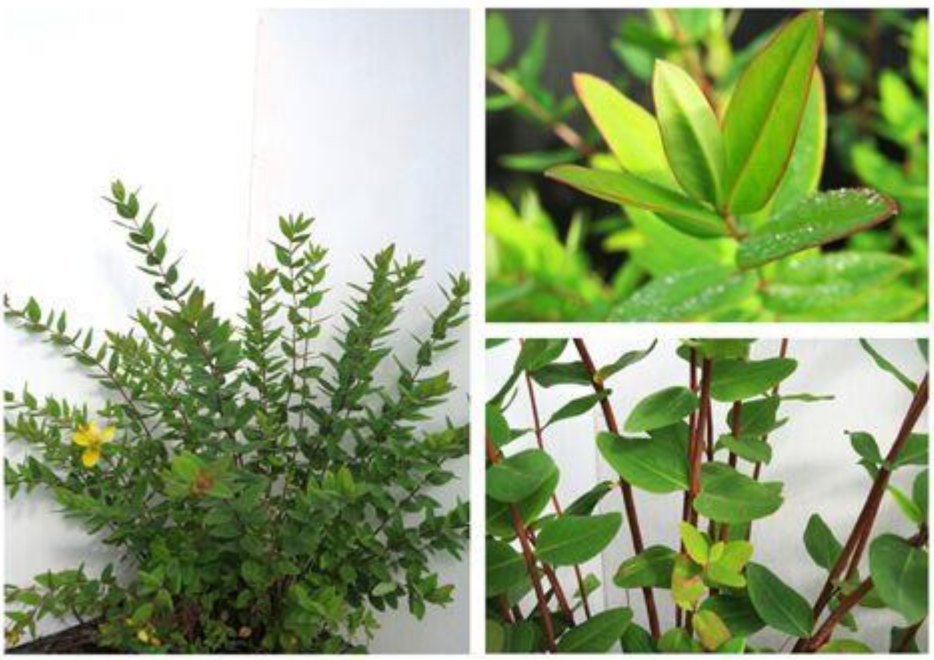

Figure S5. *Hypericum hookerianum*

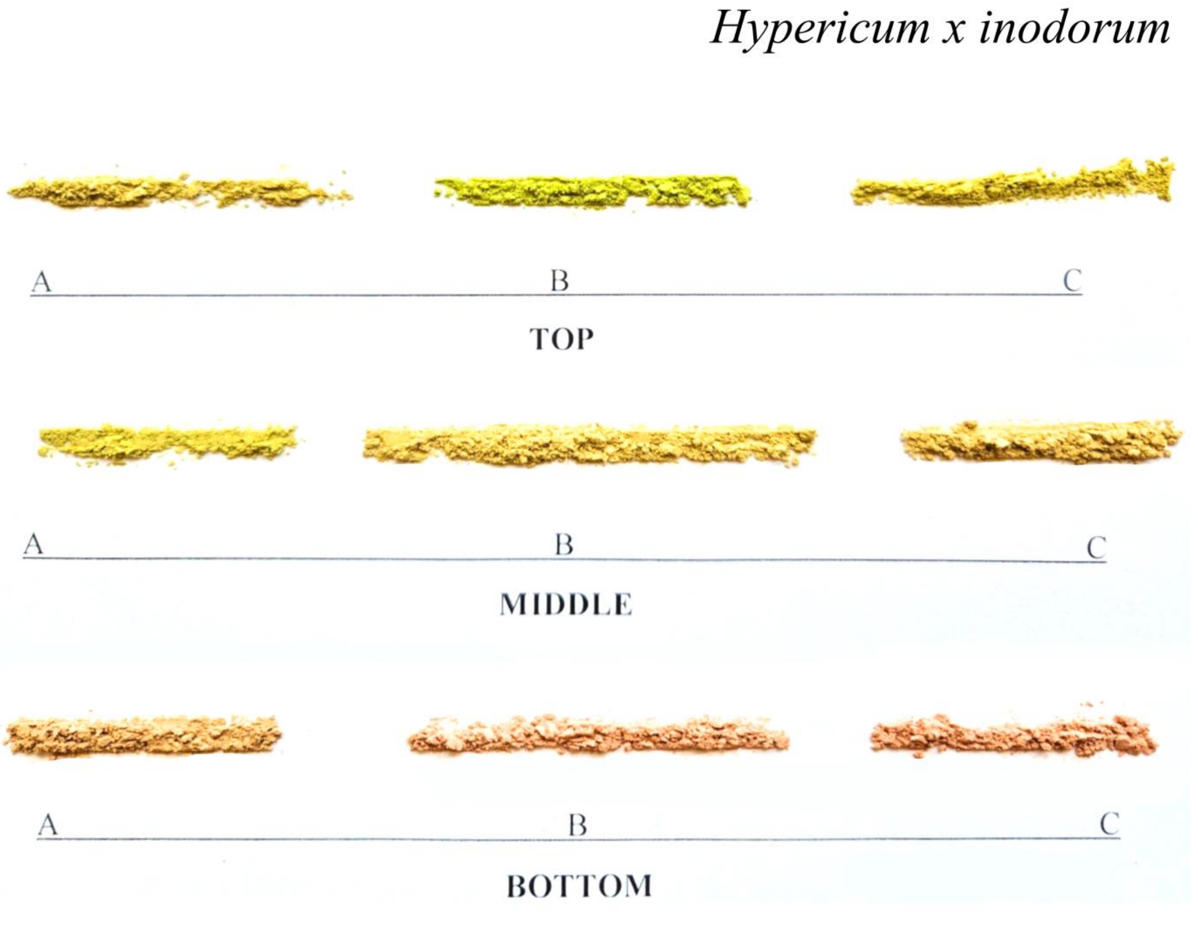

Figure S6. The powdered samples of top, middle, and bottom leaves of *Hypericum* × *inodorum*.

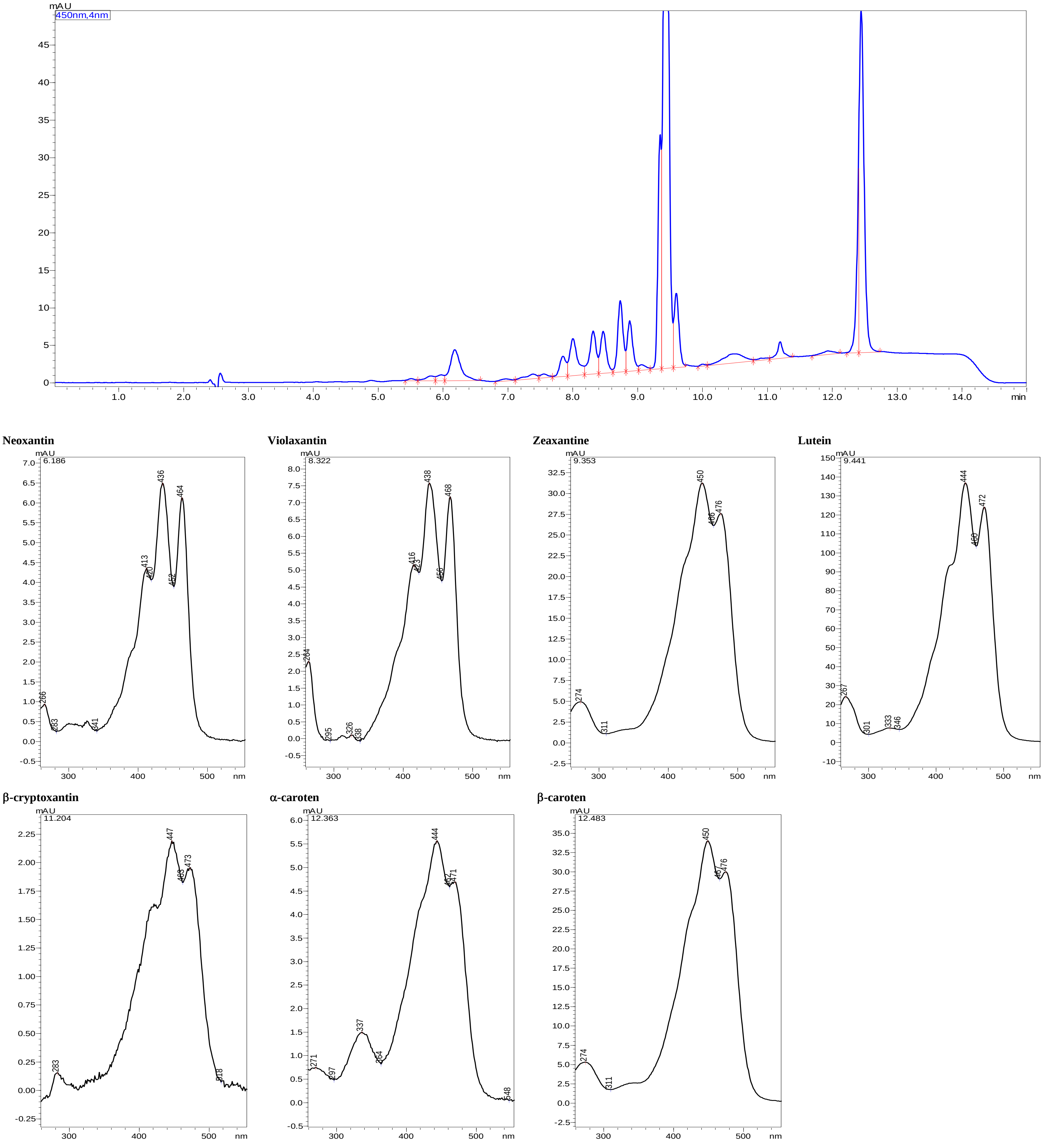

Figure S7. Chromatogram of carotenoids separation of extracts from saponified samples of *H. patulum* top leaves by PFP column, together with the UV spectra of identified carotenoids and their retention times (top left corners).

Table S1. Tocochromanol content in *Hypericum* species' leaves, mg 100 g^{-1} dw.

**Species**	**Leaf position**	**Tocotrienols**				**Tocopherols**				
		**δ-T3**	**β-T3**	**γ-T3**	**α-T3**	**δ-T**	**β-T**	**γ-T**	**α-T**	**TT**
Androsaemum section										
× *inodorum*	Bottom	18.17 ± 3.25^{ab}	0.17 ± 0.06^{a}	18.40 ± 3.97^{ab}	ND	ND	0.67 ± 0.12^{b}	6.83 ± 0.21^{b}	212.80 ± 20.66^{a}	257.03 ± 15.65^{a}
	Middle	16.83 ± 1.16^{b}	0.20 ± 0.00^{a}	18.73 ± 1.20^{ab}	ND	ND	0.40 ± 0.00^{bc}	3.20 ± 0.36^{cd}	156.70 ± 11.12^{b}	196.07 ± 12.49^{b}
	Top	18.30 ± 1.04^{ab}	0.20 ± 0.10^{a}	20.07 ± 0.60^{ab}	ND	ND	0.33 ± 0.06^{bcd}	2.50 ± 0.30^{cdef}	124.60 ± 22.89^{cd}	166.00 ± 23.98^{cd}
androsaemum	Bottom	17.77 ± 2.72^{ab}	0.23 ± 0.06^{a}	16.97 ± 2.01^{abc}	ND	ND	0.63 ± 0.06^{a}	8.63 ± 1.67^{a}	210.20 ± 9.40^{a}	254.43 ± 5.32^{a}
	Middle	18.57 ± 0.59^{ab}	0.17 ± 0.06^{a}	19.00 ± 2.31^{ab}	ND	ND	0.43 ± 0.06^{b}	2.80 ± 0.26^{sde}	151.10 ± 7.45^{be}	192.07 ± 7.85^{be}
	Top	20.80 ± 1.31^{a}	0.20 ± 0.00^{a}	22.43 ± 2.30^{a}	ND	ND	0.30 ± 0.00^{bcd}	2.90 ± 0.61^{cde}	115.33 ± 7.68^{d}	161.97 ± 10.40^{d}
Ascyreia section										
hookerianum	Bottom	3.63 ± 0.21^{cde}	ND	15.27 ± 0.85^{bc}	2.17 ± 0.45^{a}	ND	0.10 ± 0.00^{e}	1.07 ± 0.12^{f}	68.77 ± 1.65^{e}	91.00 ± 1.82^{fgh}
	Middle	5.80 ± 0.36^{cde}	ND	18.23 ± 0.68^{ab}	1.03 ± 0.15^{b}	ND	0.10 ± 0.00^{e}	1.33 ± 0.57^{ef}	48.43 ± 3.39^{efg}	74.93 ± 4.08^{ghi}
	Top	4.50 ± 1.11^{cde}	ND	12.57 ± 2.86^{cd}	0.77 ± 0.06^{c}	ND	0.07 ± 0.06^{e}	2.03 ± 0.25^{cdef}	23.57 ± 1.27^{fg}	43.50 ± 5.35^{j}
patulum	Bottom	4.97 ± 0.12^{cde}	ND	16.37 ± 1.38^{bc}	ND	ND	0.20 ± 0.00^{de}	2.43 ± 0.06^{cdef}	102.97 ± 4.69^{d}	126.93 ± 4.30^{e}
	Middle	5.27 ± 0.15^{cde}	ND	19.37 ± 0.49^{ab}	ND	ND	0.10 ± 0.00^{e}	2.23 ± 0.15^{cdef}	68.10 ± 10.41^{e}	95.07 ± 10.65^{fg}
	Top	6.57 ± 0.15^{c}	ND	20.60 ± 2.10^{ab}	ND	ND	0.10 ± 0.00^{e}	3.60 ± 0.82^{c}	30.90 ± 4.01^{fg}	61.77 ± 5.16^{hij}
pseudohenryi	Bottom	2.07 ± 0.12^{de}	ND	9.63 ± 0.76^{d}	ND	ND	0.27 ± 0.06^{cd}	1.63 ± 0.15^{def}	102.37 ± 5.10^{d}	115.97 ± 4.90^{ef}
	Middle	1.87 ± 0.23e	ND	8.13 ± 0.96^{d}	ND	ND	0.10 ± 0.00^{e}	1.40 ± 0.10^{ef}	57.07 ± 4.97^{ef}	68.57 ± 5.99^{ghij}
	Top	2.37 ± 0.21^{de}	ND	8.60 ± 0.95^{d}	ND	ND	0.10 ± 0.00^{e}	1.73 ± 0.21^{def}	32.77 ± 6.20^{g}	45.57 ± 7.10^{ij}

Data are presented as means ± standard deviation of three replicates (n = 3). ND, calculated tocochromanol content < 0.0099 mg 100 g^{-1} dw. Letters denote statistically similar values within column.

Table S2. Chlorophyll content in *Hypericum* species' leaves, mg 100 g^{-1} dw

**Species**	**Leaf position**	**Chl *a***	**Chl *b***	**Total chlorophyll**	***a*/*b***
Androsaemum section					
× *inodorum*	Bottom	3.33 ± 2.05^{fg}	1.56 ± 0.82^{fg}	4.90 ± 2.87^{ef}	2.08 ± 0.20^{f}
	Middle	9.73 ± 3.10^{bcdef}	3.51 ± 1.02^{bcdef}	13.24 ± 4.11^{bcde}	2.76 ± 0.19^{e}
	Top	12.50 ± 4.03^{abcd}	4.46 ± 1.40^{abcd}	16.96 ± 5.42^{abc}	2.80 ± 0.18^{e}
androsaemum	Bottom	2.07 ± 0.38^{g}	1.03 ± 0.15^{g}	3.10 ± 0.53^{f}	2.00 ± 0.09^{f}
	Middle	7.13 ± 0.42^{defg}	2.46 ± 0.11^{defg}	9.60 ± 0.53^{cdef}	2.89 ± 0.04^{e}
	Top	7.83 ± 0.99^{cdefg}	2.78 ± 0.40^{cdefg}	10.62 ± 1.39^{bcdef}	2.82 ± 0.06^{e}
Ascyreia section					
hookerianum	Bottom	6.77 ± 2.63^{defg}	2.18 ± 0.83^{defg}	8.95 ± 3.46^{cdef}	3.10 ± 0.04^{cde}
	Middle	14.47 ± 3.50^{ab}	4.38 ± 0.95^{ab}	18.85 ± 4.44^{ab}	3.29 ± 0.16^{bcd}
	Top	17.87 ± 0.91^{a}	5.77 ± 0.45^{a}	23.63 ± 1.31^{a}	3.10 ± 0.13^{cde}
patulum	Bottom	10.53 ± 1.77^{bcde}	3.60 ± 0.41^{bcde}	14.13 ± 2.17^{bcd}	2.91 ± 0.18^{e}
	Middle	11.87 ± 1.10^{abcde}	3.38 ± 0.29^{bcde}	15.24 ± 1.39^{abcd}	3.51 ± 0.04^{ab}
	Top	14.43 ± 1.36^{ab}	4.34 ± 0.41^{ab}	18.77 ± 1.77^{ab}	3.33 ± 0.06^{bc}
pseudohenryi	Bottom	5.67 ± 0.68^{efg}	1.91 ± 0.19^{def}	7.57 ± 0.87^{def}	2.97 ± 0.06^{de}
	Middle	14.37 ± 3.16^{ab}	4.06 ± 0.88^{abc}	18.43 ± 4.03^{ab}	3.53 ± 0.02^{ab}
	Top	13.73 ± 1.04^{abc}	3.68 ± 0.32^{bcd}	17.41 ± 1.36^{abc}	3.74 ± 0.07^{a}

Data are presented as means ± standard deviation of three replicates ($n = 3$). Letters denote statistically similar values within column.

Table S3. Carotenoid content in *Hypericum* species' leaves, mg 100 g^{-1} dw.

Species	Leaf position	Neoxanthin	Violaxanthin	Zeaxanthin	Lutein	β-Cryptoxanthin	α-Carotene	β-Carotene	Other	Total carotenoids
Androsaemum section										
× *inodorum*	Bottom	0.63 ± 0.23^{f}	0.40 ± 0.17^{bcd}	1.37 ± 0.12^{c}	8.53 ± 2.74^{g}	0.17 ± 0.06^{d}	0.50 ± 0.17^{d}	2.87 ± 1.33^{e}	3.33 ± 1.04^{fg}	17.73 ± 5.87^{hi}
	Middle	1.13 ± 0.40^{cdef}	1.13 ± 0.76^{abc}	1.83 ± 0.42^{c}	14.03 ± 3.01^{defg}	0.13 ± 0.06^{d}	0.77 ± 0.29^{d}	7.47 ± 2.64^{bcde}	5.23 ± 1.10^{cdef}	31.83 ± 8.64^{efgh}
	Top	1.50 ± 0.53^{bcde}	1.23 ± 0.57^{abc}	2.00 ± 0.56^{bc}	17.67 ± 3.44^{cdef}	0.20 ± 0.00^{d}	1.10 ± 0.30^{d}	9.23 ± 2.83^{abcd}	6.17 ± 1.15^{bcde}	39.03 ± 8.54^{cdefg}
androsaemum	Bottom	0.47 ± 0.06^{f}	0.20 ± 0.00^{d}	1.70 ± 0.36^{c}	7.60 ± 0.56^{g}	0.20 ± 0.00^{d}	0.30 ± 0.10^{d}	2.33 ± 0.32^{e}	2.53 ± 0.23^{g}	15.33 ± 1.35^{i}
	Middle	0.87 ± 0.06^{ef}	0.47 ± 0.06^{bcd}	2.17 ± 0.80^{bc}	11.67 ± 0.70^{fg}	0.13 ± 0.06^{d}	0.67 ± 0.06^{d}	5.53 ± 0.42^{de}	4.07 ± 0.23^{efg}	25.50 ± 1.73^{ghi}
	Top	0.97 ± 0.06^{def}	0.37 ± 0.06^{cd}	2.20 ± 0.61^{bc}	13.20 ± 0.20^{efg}	0.17 ± 0.06^{d}	0.57 ± 0.12^{d}	6.87 ± 0.12^{cde}	4.43 ± 0.31^{defg}	28.77 ± 0.76^{fghi}
Ascyreia section										
hookerianum	Bottom	1.23 ± 0.42^{cdef}	0.50 ± 0.10^{bcd}	3.77 ± 0.91^{ab}	19.80 ± 3.03^{cd}	0.47 ± 0.06^{c}	0.67 ± 0.20^{c}	6.17 ± 2.25^{de}	6.60 ± 0.89^{bcd}	39.17 ± 5.86^{cdefg}
	Middle	1.90 ± 0.26^{abc}	1.00 ± 0.30^{abcd}	1.97 ± 0.55^{bc}	22.00 ± 3.66^{bc}	0.57 ± 0.12^{abc}	1.53 ± 0.25^{abc}	11.63 ± 3.6^{abc}	7.33 ± 0.90^{bc}	47.93 ± 5.98^{abcd}
	Top	2.40 ± 0.00^{a}	1.27 ± 0.15^{ab}	2.80 ± 0.35^{abc}	27.27 ± 0.31^{ab}	0.53 ± 0.06^{bc}	2.43 ± 0.32^{bc}	14.17 ± 0.68^{a}	8.53 ± 0.72^{ab}	59.33 ± 2.42^{ab}
patulum	Bottom	1.70 ± 0.20^{abcd}	0.67 ± 0.15^{abcd}	2.30 ± 0.20^{abc}	20.63 ± 1.36^{cd}	0.50 ± 0.00^{bc}	1.23 ± 0.32^{bc}	9.00 ± 2.12^{abcd}	6.73 ± 0.12^{bcd}	42.70 ± 4.03^{cdef}
	Middle	1.60 ± 0.20^{bcde}	0.90 ± 0.17^{abcd}	2.70 ± 0.70^{abc}	21.97 ± 2.52^{bc}	0.47 ± 0.06^{c}	1.37 ± 0.42^{c}	9.63 ± 1.01^{abcd}	7.70 ± 1.04^{nc}	46.40 ± 5.95^{abcde}
	Top	2.10 ± 0.10^{ab}	1.57 ± 0.40^{a}	4.07 ± 1.1^{a}	28.63 ± 1.69^{a}	0.63 ± 0.06^{abc}	1.73 ± 0.25^{abc}	11.63 ± 0.64^{abc}	10.77 ± 1.67^{a}	61.13 ± 5.63^{a}
pseudohenryi	Bottom	0.93 ± 0.06^{def}	0.40 ± 0.00^{cd}	2.47 ± 0.31^{abc}	18.83 ± 1.53^{cde}	0.73 ± 0.06^{a}	0.57 ± 0.06^{a}	5.60 ± 0.36^{de}	5.43 ± 0.40^{cdef}	34.90 ± 2.10^{defg}
	Middle	1.60 ± 0.30^{abcd}	0.70 ± 0.10^{abcd}	2.30 ± 0.53^{abc}	20.80 ± 2.52^{bc}	0.63 ± 0.06^{ab}	1.43 ± 0.35^{ab}	11.53 ± 2.40^{abc}	6.30 ± 0.60^{bcde}	45.40 ± 6.16^{bcde}
	Top	1.73 ± 0.15^{abcd}	0.67 ± 0.12^{abcd}	2.67 ± 0.64^{abc}	23.87 ± 0.15^{abc}	0.57 ± 0.06^{abc}	1.53 ± 0.15^{abc}	12.43 ± 1.12^{ab}	7.07 ± 0.32^{bc}	50.53 ± 1.30^{abc}

Data are presented as means ± standard deviation of three replicates (n = 3). Letters denote statistically similar values within column.

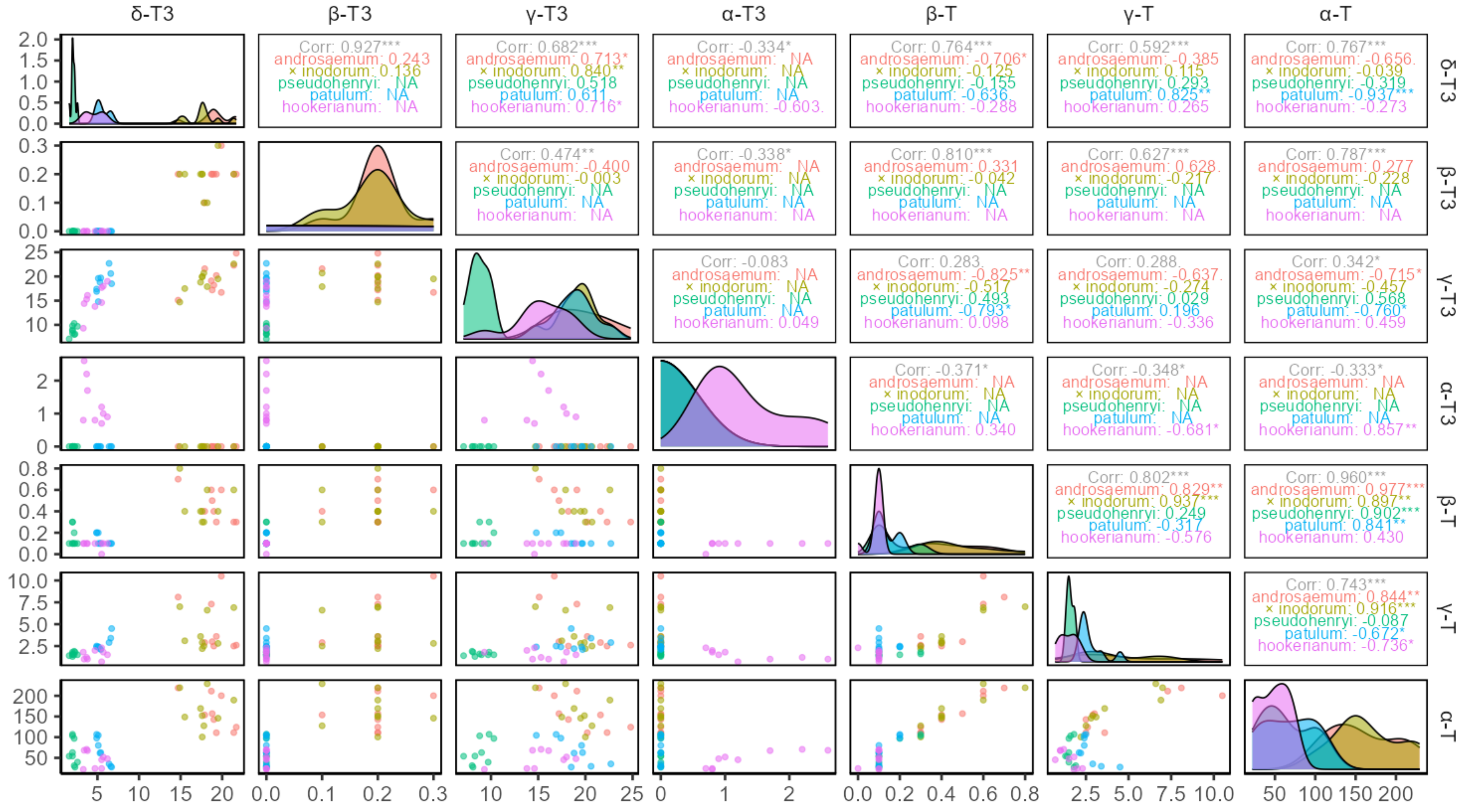

Figure S8. Spearman correlation between individual tocochromanols in *Hypericum* species' leaves and tocochromanol content density plots.

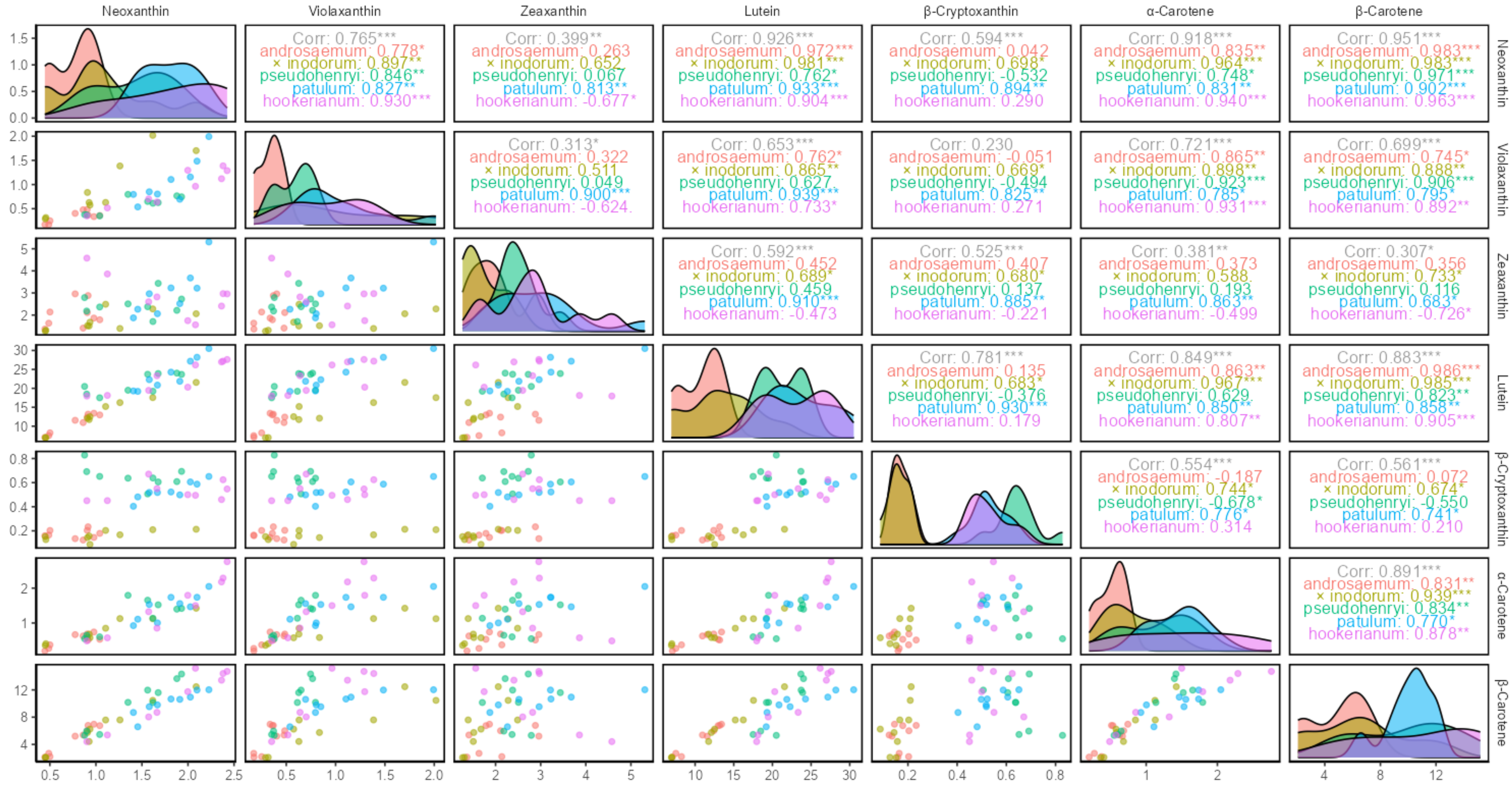

Figure S9. Spearman correlation between individual carotenoids in *Hypericum* species' leaves and tocochromanol content density plots.

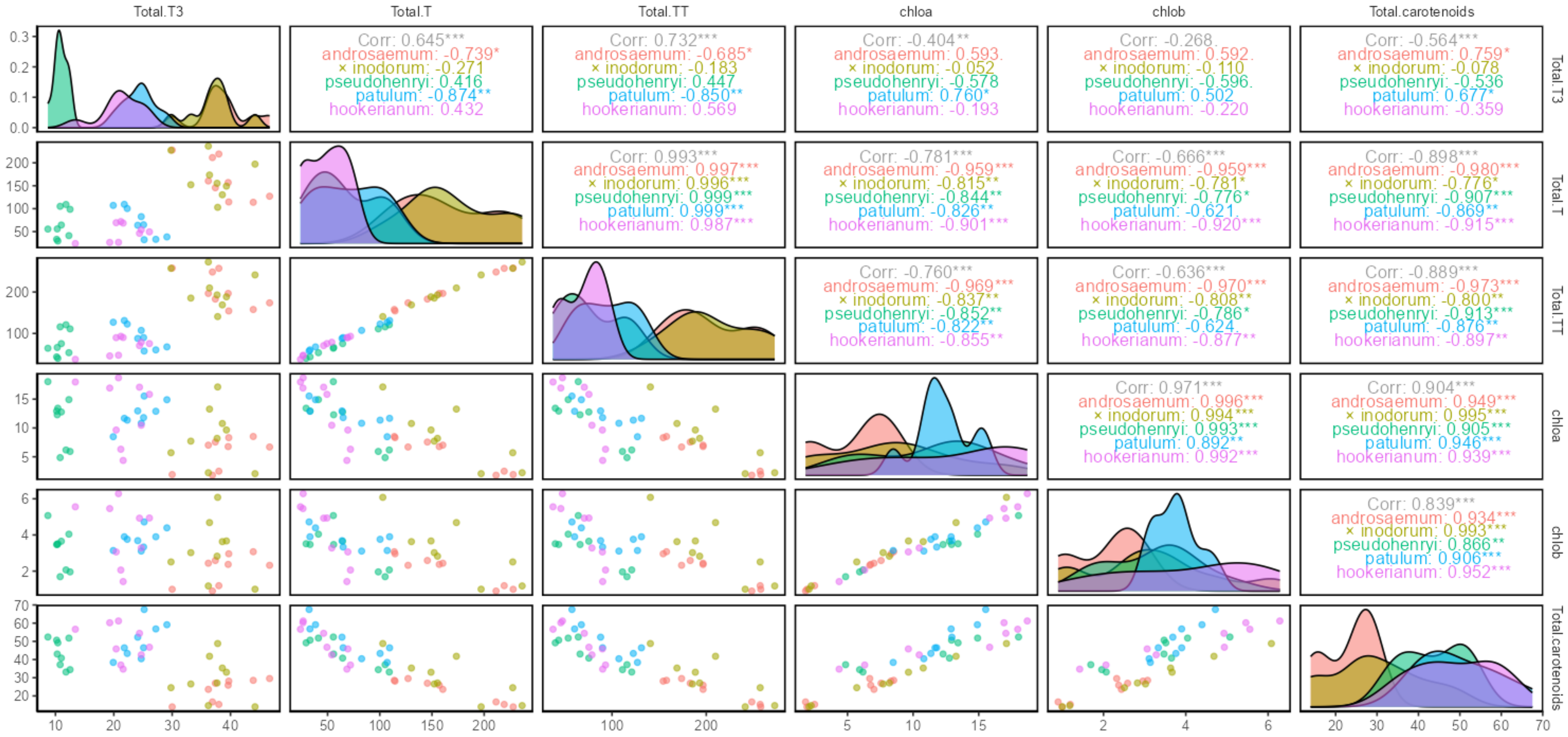

Figure S10. Spearman correlation between total tocotrienols, total tocopherols, total tocochromanols, chlorophyll *a* and *b*, and total carotenoids in *Hypericum* species' leaves and tocochromanol content density plots. Abbreviations: Total.T3, total tocotrienol content; Total.T, total tocopherol content; Total.TT, total tocochromanol content; chloa, chlorophyll *a* content; chlob, chrolophyll *b* content; Total.carotenoids, total carotenoid content.
